# Feasibility of a cardiovascular cohort in a Sub-Saharan Africa community: preliminary report of the pilot project TAHES (Tanvè Health Study) in Benin

**DOI:** 10.1080/16549716.2017.1270528

**Published:** 2017-05-15

**Authors:** Yessito Corine Houehanou, Carmelle Mizéhoun-Adissoda, Salimanou Amidou, Iléana Désormais, Martin Houénassi, Pierre-Marie Preux, Benoit Marin, Dismand Houinato, Philippe Lacroix

**Affiliations:** ^a^ U1094, Tropical Neuroepidemiology, INSERM, Limoges, France; ^b^ UMR-S 1094, Tropical Neuroepidemiology, Institute of Neuroepidemiology and Tropical Neurology, University Limoges, Limoges, France; ^c^ Faculty of Health Sciences, Laboratory of Chronic and Neurologic Diseases Epidemiology, University of Abomey-Calavi, Cotonou, Bénin; ^d^ Vascular Medicine Unit, CHU Limoges, Limoges, France; ^e^ Cardiology Unit, CNHU Cotonou, Cotonou, Bénin; ^f^ Functional Unit of Clinical Research and Biostatistics, CHU Limoges, Limoges, France; ^g^ Neurology Unit, CNHU Cotonou, Cotonou, Bénin

**Keywords:** cohort, pilot study, cardiovascular diseases, Sub-Saharan Africa

## Abstract

**Background**: Faced with the growing burden of cardiovascular disease (CVD) including atherosclerotic in Sub-Saharan Africa (SSA), the development of appropriate prediction tools, based on large cohorts, appears useful for prevention.

**Objective**: The objective of the pilot project TAHES (Tanvè Health Study) was to explore the feasibility of a large cohort study focused on CVD and risk factors in Benin.

**Methods**: We implemented a prospective cohort over 2 years. The sample consisted of all people aged 25 years or older who had lived for at least the previous 6 months in the villages of Tanvè or Dékanmè. At baseline in February 2015, behaviours and medical histories were recorded using a standardized questionnaire adapted from the WHO Steps instrument; screening questionnaires for angina, claudication, congestive heart failure, and stroke were applied; anthropometric measures and fasting capillary blood glucose were taken. All participants were included in the follow-up phase. Surveillance of target CVD and deaths was implemented through a medical and a community network.

**Results**: A total of 1,195 participants were enrolled at baseline; women represented 65.5% and the median age was 39 years. The high participation rate (91.4%), the quality of baseline data, and the functionality of the events surveillance network over 8 months indicated good perspective for the feasibility of a large cohort. We recorded a 3.8% prevalence of daily smoking, 3.6% of harmful use of alcohol, 10.7% of obesity, 25.5% of high blood pressure, and 3.5% of diabetes. Prevalence of angina pectoris (2.7%), intermittent claudication (2.0%), congestive heart failure (0.9%), and stroke survival with motor impairment (3‰) were also recorded. Ten deaths occurred during the first 8 months, all within households; a cardiovascular cause was suspected in four cases.

**Conclusion**: These preliminary results support the feasibility of establishing a cohort in Benin. It would require technical and resource support.

## Background

Cardiovascular diseases (CVD) are the leading cause of death worldwide. In 2012, an estimated 17.5 million people died from CVD, which accounted for 31% of all global deaths; 80% of these deaths occurred in low- and middle-income countries [1]. CVD such as coronary heart disease (CHD), stroke, and peripheral artery disease (PAD) are mainly due to atherosclerosis. Their increase is related to lifestyle changes and ageing. The most important behavioural risk factors are tobacco use, lack of physical activity, unhealthy diet, and harmful use of alcohol. These factors contribute to obesity, hypertension, diabetes, and lipid disorders.

Sub-Saharan Africa (SSA) has a high burden of both infectious and chronic diseases [1–3]. CVD deaths represent approximately 8.8% of total deaths, 3.9% of years of life lost, and 3.5% of DALYs (Disability Adjusted Life Years) [4]. Stroke is the main cause of CVD deaths [4]. CVD has progressed in SSA [5,6]; coronary heart diseases are not infrequent [7–9]; stroke incidence varies from 35 to 108/100,000 person-years [10–12]. Asymptomatic PAD prevalence was estimated at around 10% [13–15]. Furthermore, these disorders arise among younger subjects than in other regions [4,6,9,16]. SSA presents the highest prevalence of hypertension in the world [17].

Major cardiovascular events can be prevented by early detection and management of people at high risk. Cardiovascular risk scales have been developed from Western cohorts such as the Framingham study [18] or the SCORE project [19]. The performances of these scales could vary, depending on the source population [20–22]; adaptations of Framingham scales for other populations were proposed by some authors in Europe [23,24]. However, no large community-based cohort study was conducted in SSA to validate or to calibrate them. In addition, there is an international debate about whether the cut points for obesity metrics should be different for some ethnic groups. Risk scales in SSA should be appropriate and easy to apply by auxiliary health workers. There is a clear need for cohort studies focused on CVD in SSA [25,26]. These studies involve stability and support of the population, adapted methodology, and high financial resources; pilot cohorts help to test the methodology and feasibility of a cohort survey and appear mandatory in SSA.

The TAHES (Tanvè Health Study) project aims to develop an appropriate screening tool to identify people at risk of CVD in Benin, applicable to other similar populations in SSA.

The objective of the pilot TAHES project was to explore the feasibility of a large cohort study focused on CVD and risk factors in Benin.

## Methods

### Overview of TAHES project

We planned a cohort focused on CVD in Benin, for at minimum 4 years. Benin is a West African country of around 10 million inhabitants according to the 2013 census [27]. The commune ‘Agbangnizoun’ is selected for the study. It comprises 50 villages with approximately 30,000 target people. It is a rural population with few migrations.

The study concerns adults aged 25 years and older. A reasoned selection of two neighbourhoods was made.

The sample size calculation was founded on the expected cumulated incidence of events in 4 years (1.2%, based on a review of literature [10–12,28]), the alpha risk (5%), the beta risk (20%), and the relative risk interesting to detect (1.75) in relation with a risk factor present among 20% of people recruited. A number of 11,080 people to be included and followed was estimated.

Target events are coronary disease, stroke, congestive heart failure, major vascular amputations, and deaths. The definitions of the suspect, likely, and certain cases of each target CVD have been specified.

Demographic and lifestyle data, histories of CVD, of hypertension, and of diabetes are collected at baseline. Anthropometrics parameters, ankle brachial index (ABI), and capillary blood glucose are measured. The electrocardiogram recording is planned.

**Figure 1.**
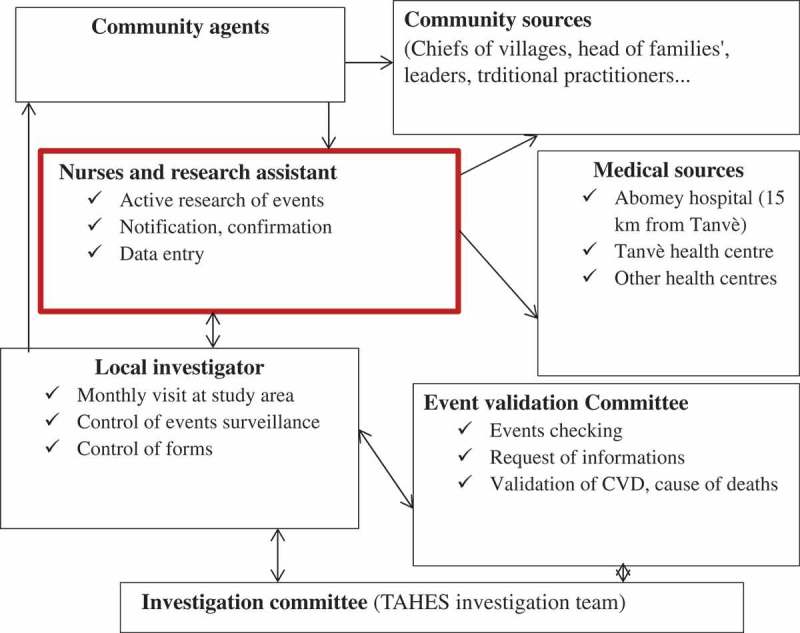
Events surveillance design in the TAHES project.

The same evaluations are done at two follow-up visits. Events surveillance outside visits is active and continuous through a network of health centres and community sources. The activities are supervised monthly by a physician member of the investigation committee. The scheme of the surveillance is presented in Figure 1.

Data are entered in an online secured database by a research assistant using a digital tablet. The database has been created and managed in collaboration with laboratory INSERM UMR 1094 NET (Tropical Neuroepidemiology, Limoges, France). It is created thanks to the Capture System software of the Clinsight company. Capture System is software for management of clinical data using the ORACLE database. The structure and the appearance of the database are unchanged compared with the data collection form.

It is accessible online thanks to a personal access (identifier and password). It is stored on the server of CEBIMER (Unit of Clinical Research, CHU Limoges, Limoges, France). Queries are performed by a data manager and data validated before analysis.

### Area, design, and study population of the pilot project TAHES

The villages of Tanvè and Dékanmè, located 300 km north of the economic capital of Benin, Cotonou, were selected for the pilot study. There is a public health centre managed by a nurse. A preliminary census was conducted in the survey area. The number of adults aged 25 and older was estimated to be 1,308.

We initiated a prospective, open cohort over 2 years. All the residents (≥ 6 months) in the study area aged 25 years or older were eligible. Pregnant women and people with major mental deficiency were not included.

### Baseline data collection

For the present study, data were collected either in households or in the public health centre of the study area in February 2015. Written informed consent was obtained from each participant. A standardized questionnaire adapted from the World Health Organization (WHO) STEPS instrument [29] was used. It included demographic, socioeconomic, and behavioural data, non-communicable disease histories, and screening questionnaires for angina pectoris [30], stroke [31], claudication [32], anxiety and depression (during the previous 12 months) [33], and congestive heart failure symptoms [34]. A French version was provided and translated into ‘Fon’, the main local language, by two professional translators. At 1 STEP, individual interviews were conducted either in French or in the local language by trained interviewers. At 2 STEP, weight, height, waist circumference, and blood pressure were measured. Weight and height were measured using mechanic materials (Seca, Germany). Blood pressure was measured in both arms by nurses, using an electronic device (Spengler, France), with adequate cuffs for normal and large arms. It was measured three times at 5-minute intervals, in seated position after a rest of at least 15 minutes. The average of the two last values was used to define blood pressure. Breathlessness, lower limb oedema, and motor impairment were sought. At 3 STEP, fasting blood glucose was measured by finger prick using a glucose meter (Accuchek Performa, Roche Diagnostics, Switzerland) in the morning after a 12‐hour overnight fast (either the same day as 1 and 2 STEP or the day after).

### Follow-up

All participants were included in the follow-up phase. Those people who leave the area are lost from follow-up. New residents, people who reach the age of 25 years, and women having given birth after a visit are included during the next visit. A network of events surveillance was implemented, involving health centres and community sources. Active and continuous research of events is conducted, based on definitions of suspected and likely cases. A weekly review of care and hospitalization registers at the health centre is done by a trained nurse. In the community, a weekly visit to heads of family, traditional practitioners, and church leaders is conducted by a community agent; a register of death and sickness in the cohort is filled. For every event, a notification form is completed within 24–48 hours and sent to the local investigator (cardiologist). Confirmation and detailed information about notification are provided by the nurse within 7 days. Verbal autopsy is applied for community death, using the WHO French version 2009 [35] within 21 days. Data entry and transmission are done locally by a research assistant. The activities are supervised by the local investigator during a monthly visit. All forms are checked and validated by the local investigator and sent to the physician members of the project. Causes of death are assigned by them. The first annual visit of participants similar to the baseline evaluation was done in April 2016 and the second is planned for April 2017.

### Definitions

Stroke was defined according to WHO criteria [36]. Congestive heart failure was defined by the presence of typical symptoms confirmed by a physician. CHD was defined by a positive WHO Rose questionnaire or a history of myocardial infarction based on medical data (the presence of typical Q waves on an electrocardiogram or acute coronary syndrome ST+ or ST– with a specific enzymatic rise). Lower limb amputation of vascular origin was defined by non-traumatic amputation of leg or thigh related to PAD based on medical data; PAD was defined by an ankle brachial index ≤ 0.9 or ≥ 1.4 or vascular stenosis at echography. Presence of claudication was suspected by a positive Edinburgh questionnaire. CVD deaths were defined by deaths arising within 28 days after a first appearance, a recurrence, or a worsening of CVD, or sudden deaths or deaths of unknown origin without other evident cause than cardiovascular; in the last case, traumatic, infectious, or tumoral causes must be excluded. Sudden death is defined by death of delay ≤ 1 hour in a subject in apparently good health.

Low fruit and vegetable intake was defined by the consumption of less than 400 g of fruits and vegetables per day. Sedentary behaviour was defined by prolonged sitting or sleeping most of the time, ≥ 8 hours per day. Harmful use of alcohol was defined by consumption of ≥ 3 drinks per day. High blood pressure was defined by systolic blood pressure ≥ 140 mm Hg or diastolic blood pressure ≥ 90 mm Hg in one of the two arms, or by currently receiving medication. High blood glucose was defined by capillary whole blood glucose ≥ 6.1 mmol/L or currently receiving medication.

### Feasibility criteria

The feasibility criteria are: (1) participation rate at baseline and quality of data, (2) number of events recorded at visits compared with number of events recorded through the continuous surveillance network, (3) percentage of people still followed-up in the cohort after 2 years.

### Ethics statements

The study was approved by Benin’s national ethics committee for health research. Agreements of the local authorities were obtained. Confidentiality is assured at all times during data collection and analysis.

### Coordination

The main investigator of the project is a head of the neurology department and director of LEMACEN in Benin. Co-responsible for the project is a chief of the vascular medicine unit of CHU Limoges, a member of INSERM UMR 1094 NET. A local investigator grants 25% of their working time to the project. A PhD student works full-time on the project. Two committees were created. The investigation committee is responsible for study implementation. The scientific committee, comprised of professors in neurology, cardiology, and public health, is responsible for methodology and events validation. Collaborations with two other French teams are planned for the enlarged project.

The team of INSERM UMR 1094 NET has already developed common projects in Benin on epilepsy, cognitive disorders, and stroke. They have already conducted a follow-up study on cognitive disorders among an elderly population in the Republic of the Congo.

### Data analysis

The analysis for this paper were limited to validated data by the time the paper was written. It concerns baseline data and the first 8 months of follow-up.

We used Epi Info 7 to analyze data for this report. Continuous variables were described using the median and the interquartile range. Qualitative variables were described using frequencies and percentages. Chi-square tests or Fisher’s exact test were used to compare proportions between groups. The crude death rate was calculated.

## Results

There were 1,308 individuals aged ≥ 25 years eligible in the area and 1,195 accepted to participate (91.4%); 27 refusals were recorded; 70 people were travelling; and 16 were interned in a traditional convent. Women predominated (65.5%). The median age was 39 years (interquartile range: 30.5–55.5). Two thirds of participants (66.4%) had no academic qualifications (Table 1). Four fifths (82%) were married or lived in a couple and nearly two thirds (62.5%) had an average household monthly income lower than the official minimal salary of 70USD.

**Table 1.** Demographic and socioeconomic characteristics of participants, pilot TAHES, Benin, 2015.

Variables	N = 1,195 n (%)
Sex (female)	783 (65.5)
Age (years)	
25–34	448 (37.5)
35–44	258 (21.6)
45–54	188 (15.7)
55–64	124 (10.4)
≥ 65	177 (14.8)
School education (years)	
None	793 (66.4)
1–6	287 (24.0)
7–10	89 (7.5)
11–13	17 (1.4)
University	9 (0.7)
Marital status	
Married/living in couple	980 (82.0)
Never married	63 (5.3)
Separated, divorced, widowed, or other	152 (12.7)
Occupation	
Farmer	240 (20.1)
Craftsman	267 (22.3)
Trader	501 (41.9)
Employee	53 (4.4)
Student/apprentice	21 (1.8)
Other	113 (9.5)
Household average monthly income (USD)	
< 70	747 (62.5)
55–140	319 (26.7)
140–210	93 (7.8)
> 210	36 (3.0)

Some criteria indicated good perspective for large cohort feasibility: a high participation rate (91.4%), quality of data collection during the baseline visit, and functionality of the events surveillance network to identify the deaths. The main barrier was the lack of medical information about deaths which occurred in households and illnesses in the cohort.

We recorded 3.8% prevalence of daily smoking, 89.5% of low fruit and vegetable intake, and 25.3% of sedentary behaviour (Table 2); 8.1% consumed alcohol at least 4 days a week, and 3.6% consumed harmful amounts. Levels of obesity (10.7%), high blood pressure (25.5%), and high blood glucose (3.5%) were also recorded. Anxiety was suspected among one fifth of participants (19.9%) and depression among one eighth.

**Table 2.** Cardiovascular diseases and risk factor distribution, pilot TAHES, Benin, 2015.

Variables (Yes)	All (N = 1195)	Men (N = 412)	Women (N = 783)	*p*
	n (%)	n (%)	n (%)	
CVD risk factors				
Alcohol consumption > 3 drinks/day	43 (3.6)	24 (5.8)	19 (2.4)	0.003
Daily tobacco smoking	45 (3.8)	34 (8.2)	11 (1.4)	< 0.001
Low fruit and vegetable intake < 400 g/day	1065 (89.1)	362 (87.9)	683 (89.8)	ns
Sedentary behaviour (≥ 8 hours/day)	303 (25.3)	153 (37.1)	150 (19.2)	< 0.001
Anxiety (during previous 12 months)	238 (19.9)	58 (14.1)	180 (23.0)	< 0.001
Depression (during previous 12 months)	156 (13.0)	49 (11.9)	107 (13.7)	ns
Obesity (BMI ≥ 30 kg/m^2^)	128 (10.7)	19 (4.6)	109 (13.9)	< 0.001
High blood pressure	305 (25.5)	97 (23.5)	208 (26.6)	ns
Fasting blood glucose ≥ 6.1 mmol/l or current medication	43 (3.6)	22 (5.3)	21 (2.7)	0.02
CVD				
Angina	32 (2.7)	9 (2.2)	23 (2.9)	ns
Intermittent claudication	24 (2.0)	3 (0.7)	21 (2.7)	0.01
Congestive heart failure	11 (0.9)	0 (0.0)	11 (1.4)	0.02
Stroke survivors with motor impairment	3 (0.3)	1 (0.2)	2 (0.3)	ns

Notes: BMI: Body Mass Index; fasting high blood glucose: fasting capillary glucose ≥ 6.1 mmol/l or currently on medication; high blood pressure: systolic blood pressure (SBP) ≥ 140 mmHg or diastolic blood pressure (DBP) ≥ 90 mmHg or currently on medication; N: total number; n: number; ns: non-significant; *p*: *p*‐values indicate the differences between men and women.

Prevalence of angina pectoris (2.7%), intermittent claudication (2.0%), congestive heart failure (0.9%), and stroke survivors with motor impairment (3‰) were also estimated.

Ten deaths, all occurring in households, were notified over 8 months. The crude death rate was 12.5/1,000 person-years. Causes of death were undetermined in two cases. CVD were suspected in four cases (two sudden deaths and two cases of congestive heart failure). The four other cases were deaths of infectious origin. No new case of non-fatal target CVD was identified; during the period, we identified no cases of hospitalization or care in the local resources for CVD events.

## Discussion

The study showed a high participation rate among the source population. People were informed about the follow-up in the study and accepted to participate. They gave their agreement and written consent separately for baseline evaluation and follow-up. The acceptability of follow-up does not seem to be a problem. Previous studies have shown high participation rates at baseline of cohort studies [37,38]. High follow-up rates at visits from 68 to 91% were also reported [38–40]. Inclusion of more individuals in prospective cohort studies in SSA appears possible. The choice to collect follow-up data in households could improve people retention in our study. Use of mobile phones could be integrated into our study, particularly in terms of the continuous surveillance. Use of mobile phones was experimented with in a cohort follow-up in SSA and appeared useful [38]. It could be a good communication tool for maintaining contact with families.

We noted high levels of high blood pressure (25.5%) and obesity (10.7%), similar to Benin’s national STEPS survey results in 2008 [41].

The crude death rate was 12.5/1,000 person-years. All deaths occurred in households; people who died did not receive medical care at the time of death. They did not attend health facilities at the time of their deaths nor during their sickness. The crude death rate in this study is close to those reported from 22 demographic surveillance sites among people ≥ 15 years old in Africa and Asia (10.8/1,000 person-years) [42]. In 2010, the age-standardized death rate (WHO standard population) of CVD was estimated at 8.9/1,000 person-years in SSA [4].

We recorded four cases of deaths probably of cardiovascular origin. This proportion is important and needs to be confirmed by further results. However, this result does not seem to be specific to our study; a high proportion of cardiovascular deaths was previously reported in a South African cohort [39].

CVD causes were based on verbal autopsy and could have been overestimated in our study. Cause-of-death data from SSA are usually not known from death certificates; the high frequency of community deaths was as previously described [43]. This situation underlines the low level of health care requests in rural areas; it could be explained by social representations of illness and the use of traditional medicine.

The network of events surveillance is functional for recording deaths. The ascertainment of medical conditions is the major challenge. No incident of non-fatal CVD was registered, probably due to default case identification in communities. Strengthening of community networks and health promotion interventions should improve requests for health care and chronic disease surveillance in our study and elsewhere in SSA. Interventions should take into account low levels of instruction, poverty, and social beliefs in rural populations. Besides, health systems should be improved. Visits appear important in this context and are useful for collecting medical data. It appears important to maintain two visits in 4 years as we planned for the TAHES project.

Another limitation of the pilot study concerns CVD ascertainment at baseline. CVD are self-reported or based on screening questionnaires. These conditions could have been over- or under-estimated. Not all data planned in the TAHES project were collected in the pilot study taking into account the funding and variables useful for feasibility criteria assessment; electrocardiogram and ankle brachial index were not recorded. We plan to complete these data in April 2017.

The assessment of feasibility in this paper is partial. The feasibility of the TAHES project will be completely assessed at the end of the pilot study. However, these preliminary results could allow us to improve the methodology of the TAHES project. They could inform other investigators and guide policies in the study area and other SSA rural areas. They could also support the search for financing for the expanded project. The cost of the pilot study is estimated at 60,000€ for 2 years or approximately 25€ per person per year. The funding of the pilot study has been achieved. The cost of the expanded cohort for 4 years is estimated at approximately 20€ per person per year. The search for funding is in progress.

The small sample size in the pilot study is a major limitation. This size is not compatible with precise estimates. It might have not allowed sufficient power to detect any significant differences between groups. Nevertheless the primary aim of this pilot study was to assess the feasibility of a larger cohort. The size of the TAHES cohort will be around 11,000 people; according to our estimation, 120 events will be recorded over 4 years. As attritions and deaths during follow-up might reduce sample size, this cohort will be dynamic.

A large cohort study in SSA seems to be technically feasible despite health system weakness and the challenge of ascertaining medical conditions. The study could be expanded after the pilot period.

## Conclusion

Preliminary results of the TAHES pilot project show good prospects for cardiovascular cohort feasibility in SSA. Strengthening the content of visits and the community network for target events’ surveillance could improve ascertainment of medical conditions in CVD cohorts in SSA rural areas. Establishing a cohort in Benin would require technical and resource support.

## Paper context

The growing CVD burden in SSA highlights the need for large cohorts to establish appropriate prediction tools to prevent these diseases. We initiated a pilot cohort in a rural area to test the feasibility of a large CVD cohort in Benin. The first results showed good study acceptability and surveillance of deaths. The ascertainment of CVD outside visits is the challenge. It could be improved by periodic visits and strengthening of the events surveillance scheme. A large cohort seems feasible. It would require technical and financial support.
